# From Dismissal to Partnership: Patient Experiences of Recurrent Urinary Tract Infection Healthcare Informed by the Theoretical Domains Framework and Behaviour Change Theory

**DOI:** 10.1111/hex.70629

**Published:** 2026-03-01

**Authors:** Abigail F. Newlands, Melissa L. Kramer, Sarah Snuggs, Katherine A. Finlay

**Affiliations:** ^1^ School of Psychology and Clinical Language Sciences University of Reading Reading Berkshire UK; ^2^ Live UTI Free Ltd Dublin Ireland

**Keywords:** behaviour change, healthcare quality, patient experience, patient‐doctor communication, qualitative, recurrent urinary tract infection, shared decision‐making

## Abstract

**Background:**

Recurrent urinary tract infection (rUTI) is common, debilitating, and associated with substantial negative impact on quality of life. Despite this, rUTI healthcare is often experienced as fragmented, dismissive, and poorly aligned with patient needs. Applying behavioural science theory to systematically identify modifiable intervention targets offers a promising but unexplored approach to improving rUTI care.

**Objective:**

To explore patient experiences of rUTI healthcare in the United Kingdom, identify barriers to and facilitators of quality care, and generate theory‐informed targets for behaviour‐change intervention and service improvement.

**Design:**

Qualitative interview study using reflexive thematic analysis, followed by deductive mapping of themes to the Theoretical Domains Framework (TDF), Behaviour Change Technique Taxonomy (BCTT), and Behaviour Change Intervention Ontology (BCIO).

**Setting and Participants:**

Semi‐structured one‐to‐one interviews with 26 adults living with rUTI in the United Kingdom, with the interview schedule informed by the TDF.

**Results:**

Four barrier themes revealed systematic challenges: ‘*Struggling with the System*,’ ‘*Unheard Voices*,’ ‘*Shouldering Blame*’ and ‘*Forced to Become an Expert*.’ Together, these captured how diagnostic limitations, fragmented services, clinical dismissal, and individualised blame compel people living with rUTI into self‐advocacy experienced as exhausting. Four facilitator themes demonstrated that quality care is achievable: ‘*Feeling Validated*,’ ‘*Partners in the Puzzle*,’ ‘*Continuity and Connection*’ and ‘*Expanding the Toolkit*.’ All 14 TDF domains were implicated, most frequently ‘social influences,’ ‘beliefs about consequences,’ ‘environmental context and resources’ and ‘knowledge,’ indicating improvement requires both system restructuring and interpersonal skill development. Mapping to the BCTT and BCIO identified specific intervention techniques targeting these domains.

**Conclusion:**

People living with rUTI face structural and relational challenges in healthcare that compound illness burden. When individuals feel believed, involved, and supported, rUTI healthcare experiences are transformed. By integrating reflexive thematic analysis with behavioural theory, this study demonstrates that improving rUTI care requires attention to both system‐level factors such as diagnostic flexibility, service continuity, and treatment options, alongside relational factors, particularly validation and shared decision‐making. These findings provide a theoretically grounded foundation for intervention development, with broader relevance for chronic conditions characterised by diagnostic uncertainty.

## 1 Introduction

Recurrent urinary tract infection (rUTI), defined as two or more infections within six months or three within a year [1], affects approximately 100 million people worldwide annually [2]. Beyond acute urinary symptoms and pain [3, 4], rUTI is associated with a substantial and enduring impact on quality of life that is often under‐recognised in clinical practice [5, 6, 7, 8, 9, 10, 11]. People living with rUTI report psychological distress and reduced wellbeing, including anxiety about recurrence, depressive symptoms, and sleep disruption [5, 6, 12, 13, 14]. Repeated infections and persistent symptoms interfere with work productivity and participation, contributing to financial strain and uncertainty [8, 9, 13, 15]. In addition, rUTI significantly affects intimacy and relationships, with up to 78% of those living with rUTI experiencing sexual distress [8, 16].

Despite its prevalence and impact, people living with rUTI frequently encounter difficulties when seeking care. Qualitative research has documented experiences of dismissive clinical encounters, a perceived lack of clinician empathy and understanding of rUTI, and limited access to longer‐term management strategies [5, 9, 11, 17]. These experiences are compounded by established limitations of standard diagnostic tools: dipstick urinalysis and standard urine culture (SUC) demonstrate notable false‐negative rates in symptomatic individuals, with SUC missing up to 58% of true infections in people with rUTI [18, 19, 20, 21, 22, 23]. Despite this known insensitivity, negative results are commonly used to dismiss patient‐reported symptoms [21, 24, 25]. For people living with rUTI, this creates a mismatch between lived experience and clinical assessment that undermines trust and the patient‐clinician relationship. Beyond the immediate impact on people living with rUTI, delayed or inadequate treatment of UTI episodes can result in serious complications including pyelonephritis and sepsis [26, 27, 28, 29]; outcomes potentially preventable with timely management.

In the United Kingdom, people living with rUTI typically seek care through general practice, where GPs make prescribing decisions, or increasingly through community pharmacists under schemes such as the NHS Pharmacy First service [30, 31]. Secondary care referral to urology or urogynaecology is often dependent on local commissioning and referral criteria such as complicated presentations or repeated infections without known causes [31]. However, evidence indicates that people living with rUTI often experience challenges accessing these services in routine practice [5, 17, 32, 33]. Similar patterns exist internationally, with primary care often managing rUTI, though healthcare pathways, diagnostic approaches, and prescribing autonomy vary considerably across settings [1, 34, 35].

Further complicating rUTI management, antimicrobial resistance (AMR) concerns create tensions between antimicrobial stewardship priorities and the need for effective symptom relief [11, 17, 33, 36, 37, 38]. While reducing unnecessary antimicrobial exposure is an essential public health goal, people living with rUTI often describe the consequences of delayed or withheld treatment: prolonged pain and disruption, feelings of abandonment, and increased symptom recurrence [17, 33]. Balancing population‐level risk with individual need remains challenging for both clinicians and people living with rUTI.

Understanding how people experience rUTI healthcare, including their perceptions of how healthcare systems respond to UTI recurrence, is therefore critical to service improvement. To date, qualitative research in this area has provided some important insights into healthcare dissatisfaction and unmet need, but no work has systematically examined patient experiences using theory to identify actionable targets for behaviour change and service improvement. The current study addresses this gap by exploring patient experiences through the lens of the Theoretical Domains Framework (TDF), a comprehensive, integrative framework developed to understand behaviours and inform intervention development in healthcare contexts [39]. Applying the TDF enables movement beyond description towards theoretically‐informed recommendations for improving care. Integrating behavioural theory with experiential data contributes not only to understanding what may go wrong in rUTI care, but also to exploring why known problems persist and where intervention efforts may be most effective.

This qualitative interview study therefore aimed to explore, from patient perspectives, the barriers to and facilitators of quality rUTI healthcare experiences in the United Kingdom. Additionally, by mapping findings to the TDF [39], the Behaviour Change Technique (BCT) Taxonomy [40, 41, 42], and the Behaviour Change Intervention Ontology (BCIO) [43, 44], this study aimed to identify theoretically‐driven targets for intervention and provide practical recommendations to improve experiences and care quality for people living with rUTI.

## 2 Materials and Methods

### 2.1 Study Design

This qualitative study employed reflexive thematic analysis (RTA) to explore patient experiences of rUTI healthcare [45] One‐to‐one semi‐structured interviews captured rich data from people living with rUTI. The interview schedule (Table 1) was informed by the TDF (v2) [39] to ensure thorough exploration of potential behavioural determinants. Open‐ended questions encouraged holistic exploration of patient experiences, with each question potentially eliciting content relevant to multiple TDF domains with a view to avoid overprescribing or narrowing interview direction and participant responses [45, 46, 47]. Analysis combined inductive and deductive approaches, attending to both semantic (explicit content) and latent (underlying meanings and implications) levels of interpretation. Initial coding and theme development were entirely data‐driven, followed by systematic mapping of themes against the TDF, BCT Taxonomy, and BCIO to identify behavioural determinants and generate theory‐informed recommendations for intervention development [39, 40, 41, 42, 43, 44]. The integration of both approaches, facilitated via application of RTA, allowed findings to be both experientially grounded and theoretically actionable. This study followed Braun and Clarke's Reflexive Thematic Analysis Reporting Guidelines (RTARG) and Big Q Qualitative Reporting Guidelines (BQQRG; see Supporting Information S1) [48, 49].

**Table 1:** Interview schedule.

Question
1. To start off, please can you describe your history with recurrent urinary tract infections and the healthcare you have experienced for this condition.
2. Can you tell me what you feel makes good communication possible when you are attending medical appointments for recurrent UTI?
3. During a medical appointment for recurrent UTI, what do you feel are the most important topics to discuss?
4. Thinking of clinicians and the ways they interact with you in medical appointments for recurrent UTI, what do you find most helpful? Why?
5. As the person receiving medical care, can you tell me about how the ways you may communicate may affect the appointment in a positive way?
6. Thinking about the environment within which you attend medical appointments for your recurrent UTI, what do you feel are the factors that encourage helpful communication?
7. Thinking back to a recent medical appointment you attended for your recurrent UTI, to what extent do you feel you shared in the decision‐making process with your doctor or other healthcare professional?
8. Can you tell me about any factors that you feel may hinder or act as barriers to you getting involved in the medical decisions made about your recurrent UTI?
9. If we were to develop a resource or tool to improve patient‐doctor communication in recurrent UTI, what do you think it should include? Why?
10. Is there anything else you would like to add?

*Note:* A full overview of the interview schedule with suggested follow‐up probes and TDF mapping is available in Supporting Information S2.

#### 2.1.1 Theoretical Domains Framework and Behaviour Change Theory

The TDF is a comprehensive synthesis of 33 behaviour change theories organised into 14 domains: ‘knowledge,’ ‘skills,’ ‘social/professional role and identity,’ ‘beliefs about capabilities,’ ‘optimism,’ ‘beliefs about consequences,’ ‘reinforcement,’ ‘intentions,’ ‘goals,’ ‘memory, attention and decision processes,’ ‘environmental context and resources,’ ‘social influences,’ ‘emotion’ and ‘behavioural regulation.’ [39] This framework enables identification of psychological, social and environmental determinants underlying behaviour.

`Once TDF domains are identified, they can be systematically linked to Behaviour Change Techniques (BCTs), the active components of interventions, using established matrices [40, 41, 42]. The Behaviour Change Intervention Ontology (BCIO) further characterises intervention content and mechanisms of action, enabling comprehensive specification of intervention components [43, 44]. Together, these frameworks support movement from identifying behavioural determinants to indicating actionable, theory‐informed intervention recommendations.

### 2.2 Participants and Recruitment

Twenty‐six adults living with rUTI in the UK were recruited via snowball sampling (see Table 2) [50]. Recruitment was predominantly achieved through sharing the study details via key UTI stakeholders, including the leading global UTI patient research and advocacy organisation, Live UTI Free (https://liveutifree.com/). Newsletter posts, website notifications, and social media posts were used, with interested individuals, other UTI and women's health organisations and UTI clinicians encouraged to share the study details.

**Table 2:** Participant characteristics.

Pseudonym	Sex assigned at birth	Age (Years)	Highest level of education	Household income	Relationship status	UTI episodes in past 12 months	Years of UTI history
Nicole	Female	40–49	Bachelor's degree	£1–£9,999	Single	7	2
Rachel	Female	40–49	Master's degree	£75,000–£99,999	In a relationship	9	4
Bea	Female	18–29	Bachelor's degree	£25,000–£49,999	Single	6	10
Sian	Female	30–39	Bachelor's degree	£100,000 or more	In a relationship	10	15
Tracy	Female	50–59	Master's degree	£50,000–£74,999	Single	5	40
Karen	Female	70+	Master's degree	£25,000–£49,999	In a relationship	3	10
Michael	Male	60–69	Secondary school	£25,000–£49,999	In a relationship	7	5
Scarlet	Female	30–39	Bachelor's degree	No current income	Single	15	13
Anna	Female	30–39	Master's degree	Prefer not to say	Single	8	17
Christine	Female	60–69	Secondary school	£75,000–£99,999	In a relationship	2	5
Vanessa	Female	30–39	Bachelor's degree	£50,000–£74,999	Single	8	7
Zara	Female	18–29	Doctoral level training	£10,000–£24,999	In a relationship	10	5
Dalia	Female	18–29	Master's degree	£25,000–£49,999	In a relationship	8	3
Paul	Male	50–59	Master's degree	£50,000–£74,999	In a relationship	3	19
Sophie	Female	40–49	Bachelor's degree	£100,000 or more	In a relationship	13	20
Ellie	Female	30–39	Bachelor's degree	£10,000–£24,999	In a relationship	5	15
James	Male	50–59	Master's degree	£100,000 or more	In a relationship	4	7
Alexandra	Female	18–29	Master's degree	£50,000–£74,999	In a relationship	5	15
Susan	Female	60–69	Bachelor's degree	£50,000–£74,999	In a relationship	7	3
Jean	Female	60–69	Some secondary school	No current income	Single	6	5
Elizabeth	Female	60–69	Bachelor's degree	£50,000–£74,999	In a relationship	6	65
Maya	Female	40–49	Secondary school	£100,000 or more	In a relationship	8	24
Julie	Female	70+	Prefer not to say	Prefer not to say	In a relationship	12	65
Charlotte	Female	30–39	Bachelor's degree	£10,000–£24,999	Single	4	11
Naomi	Female	18–29	Secondary school	£50,000–£74,999	In a relationship	11	13
Lina	Female	18–29	Bachelor's degree	£25,000–£49,999	In a relationship	6	21

*Note: N* = 26.

Eligible participants were identified via an online screening survey. Inclusion criteria were: aged 18 years or older, residing in the United Kingdom, current diagnosis of rUTI (two or more UTIs in the past six months, or three or more in the past year) [1], and the ability to participate in an English‐language interview. To maintain focus on uncomplicated rUTI presentations, participants were excluded if they had a current diagnosis of complicated UTI, interstitial cystitis, or neurogenic dysfunction of the lower urinary tract, reported urinary tract abnormalities contributory to rUTI (e.g., urinary tract calculi), or used an indwelling catheter.

Participants were aged between 24 and 70 years old (*M* = 45.7, SD = 16.6), were predominantly female (*n* = 23, 88.5%), and mostly identified as White (*n* = 22, 84.6%). Approximately two‐thirds of participants were in a relationship at the time of interview (*n* = 18, 69.2%). The average number of UTI episodes reported in the past year was 7.23 (SD = 3.20), and participants reported an average of 16.1 years of UTI history (SD = 16.7).

Sample size in RTA is not determined by data saturation calculations or predetermined thresholds, which are inconsistent with the epistemological foundations of reflexive approaches [45, 48, 51]. The final group size prioritises depth over breadth, focusing on the ability to generate rich, nuanced insights from each participant's story rather than quantitative generalisability [45, 48, 51]. Information power and meaning saturation were prioritised [45, 51], with recruitment continuing until we had achieved sufficient richness and complexity to construct meaningful themes addressing our research aims.

### 2.3 Interview Procedure and Reflexivity

A 10‐item semi‐structured interview schedule was developed (Table 1), informed by the TDF [52], existing rUTI healthcare experience and shared decision‐making (SDM) literature [5, 11, 53, 54], and consultation with a key UTI stakeholder group with expertise in the rUTI patient experience, Live UTI Free. The research team co‐developed the interview schedule with significant input from MLK and KAF, who bring both qualitative research expertise and personal experience of living with rUTI to the study. Open‐ended questions explored patient‐perceived barriers to and facilitators of communication and involvement in rUTI healthcare, with follow‐up probes encouraging elaboration and allowing participants to define what mattered most. Interview questions explored all 14 TDF domains to facilitate subsequent deductive mapping (see Data Analysis). A full overview of the interview schedule with suggested follow‐up probes and TDF mapping is available in Supporting Information S2.

Individuals with an interest in participating reviewed a participant information sheet before completing an e‐consent form and screening questionnaire. Selected participants took part in an online interview via Microsoft Teams, which were audio‐recorded to facilitate anonymised transcription. Interviewer field notes were recorded during and after interviews. On average, interviews lasted 42.7 min (SD = 10.5).

All interviews were conducted by the first author (AFN), a psychological researcher with postgraduate training in advanced qualitative methods, personal experience of UTI and related urogynaecological conditions, and established relationships with the community of people living with rUTI. This facilitated rapport building and understanding of common patient experiences. Following RTA reflexivity principles [45, 48, 49, 55], AFN maintained awareness of her own responses during interviews, including empathy with participant challenges and the desire to reassure, and used these as tools to understand more deeply participant experiences whilst upholding appropriate professional boundaries. Her personal experience of navigating healthcare for urogynaecological conditions facilitated rapport and sensitivity to experiences of dismissal, while regular analytical discussions with co‐authors ensured interpretations remained grounded in participant accounts rather than her own experiences. AFN maintained a reflexive journal throughout data collection and analysis, documenting responses to interviews, interpretations, and analytical decisions.

### 2.4 Data Analysis

#### 2.4.1 Inductive Phase: Coding and Theme Development

RTA was employed following Braun and Clarke's six‐step process [45, 48, 49]. The first author read and re‐read transcripts to achieve deep familiarity with the data, then inductively generated initial codes across the entire dataset using NVivo 14 qualitative research software. Codes represented potential features related to understanding barriers to and facilitators of quality rUTI healthcare without imposing external frameworks. Codes were applied iteratively and flexibly, capturing both semantic (i.e., explicit surface‐level) and latent (i.e., underlying conceptual) meaning. We then identified patterns of shared meaning and developed these into potential themes; candidate themes were reviewed iteratively against coded extracts and the entire dataset. Regular analytical discussions with co‐authors, bringing perspectives from patient experiences and advocacy (MLK, KAF) and health psychology practice and research (SS, KAF), provided critical dialogue to examine and refine developing themes. The final themes were organised into barriers to and facilitators of quality rUTI healthcare.

#### 2.4.2 Deductive Phase: Theoretical Mapping

Following inductive theme development, themes were mapped deductively against the TDF [39]. The first author (AFN) systematically reviewed each theme's content against TDF domain definitions using the coding manual by Atkins et al. [39], with mappings discussed with co‐authors (MLK, SS, KAF) to ensure theoretical accuracy and completeness. This provided mechanistic insight into how and why themes function as barriers or facilitators. Themes were subsequently linked to BCTs and the BCIO using established TDF‐BCT matrices [39, 41, 56] and the Theory and Techniques Tool [57], and then to corresponding BCIO classes [43] to identify intervention characteristics and potential mechanisms of action (MoAs) [40, 41, 42, 43, 44]. Finally, theory‐informed recommendations were generated by translating these mappings into actionable clinical targets, either addressing barriers or encouraging facilitators. This systematic process ensured recommendations were theoretically grounded rather than solely experientially derived [40, 41, 56, 57].

### 2.5 Ethical Considerations

This study received ethical approval from the University of Reading Research Ethics Committee (reference: UREC 24/17). All participants provided informed e‐consent before interviews, and were advised that their participation was confidential, optional, and could be withdrawn at any time without giving a reason. Pseudonyms have been employed and personal identifying information removed to maintain participant confidentiality.

## 3 Results

**Figure 1:**
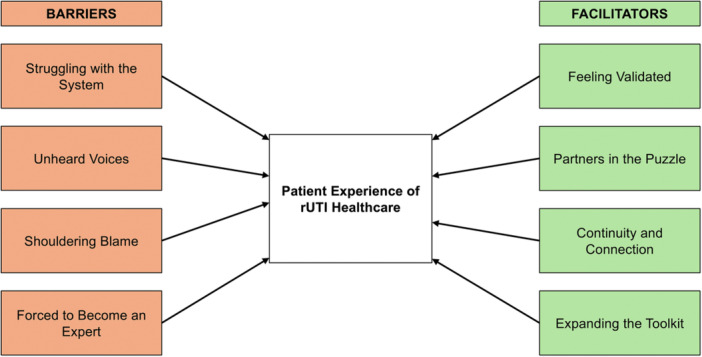
Thematic map of barriers to and facilitators of quality rUTI healthcare. Analysis identified four barrier themes (orange) and four facilitator themes (green).

Participants described living with rUTI as a prolonged struggle for recognition, continuity, and care that went beyond acute symptoms and test results. Across interviews, rUTI was framed as an ongoing disruption to everyday life, identity, and wellbeing, characterised by repeated cycles of uncertainty, distress, and exhausting self‐advocacy. Analysis identified eight themes, organised into four barriers and four facilitators (Figure 1). Themes captured both the perceived system‐level constraints and the relational and practical conditions that facilitate quality healthcare experiences.

**Figure 2:**
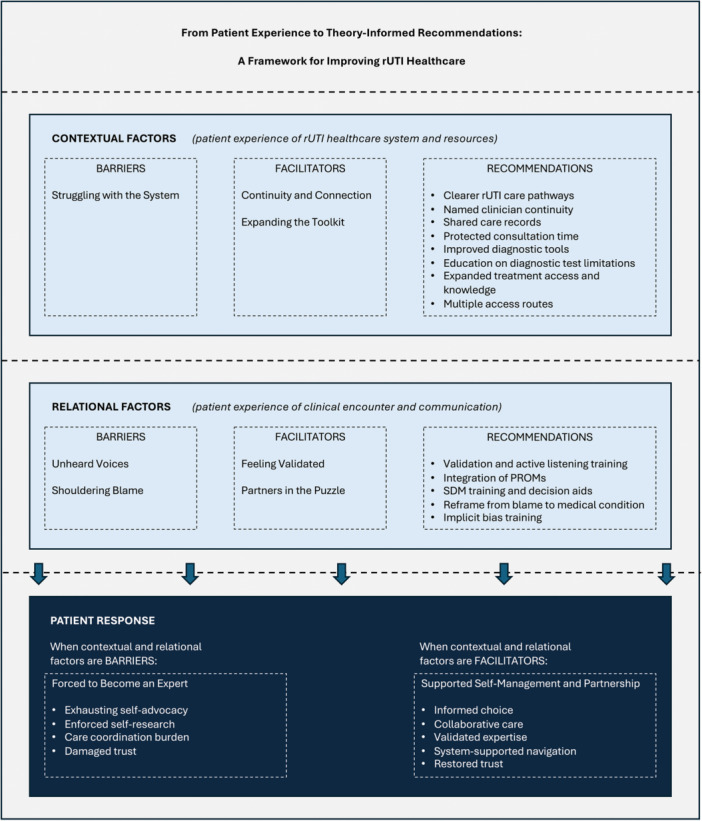
Theoretical framework for improving rUTI healthcare experiences. Patient experiences are organised across two levels of influence: contextual factors (system and resources), and relational factors (clinical encounter and communication). Corresponding barriers, facilitators, and theory‐informed recommendations are provided at each level. The patient response level illustrates how barriers at contextual and relational levels require people living with rUTI to become experts in their own care, characterised by exhausting self‐advocacy and reduced trust in the healthcare system. Themes have been mapped to the Theoretical Domains Framework (TDF) domains to identify behavioural determinants underlying patient experiences and theory‐informed recommendations. Illustrative quotations and detailed mappings with the TDF, Behaviour Change Techniques (BCTs), and Behaviour Change Intervention Ontology (BCIO) are presented in Table 3 [39, 40, 41, 42, 43, 44].

Barrier themes clustered around perceived rUTI healthcare system inadequacy, clinical dismissal, shouldering blame, and the resultant need for people living with rUTI to develop expertise. Importantly, facilitator themes demonstrated that when certain conditions were present – validation, collaboration, continuity, and expanded treatment knowledge and options – participants described markedly different and more positive healthcare experiences. Illustrative quotations supporting each theme and their theoretical mappings are presented in Table 3, with selected quotations integrated into the narrative below. The integrated theoretical framework is illustrated in Figure 2.

**Table 3:** Theoretical theme mapping and recommendations.

Theme	Illustrative quotations	TDF domains	Priority BCTs	BCIO classes (ID)	Key recommendations
BARRIERS					
*Struggling with the system*	*“The standard NHS tests are a low level of efficiency. I think the threshold is probably too high. It needs recalibrating.”* (James) *“At no point did the GP say I should get a referral. I had to request it myself.”* (Sian) *“Let's talk about* why *this keeps happening.”* (Susan) *“My worry is I'm going to be resistant to everything at some stage, but I can't not live with taking antibiotics when I get a UTI.”* (Tracy) *“It was a battle. I went to the doctor about 20 times in two months. I'm still not better after years.”* (Naomi)	Environmental Context and Resources; Knowledge; Beliefs about Consequences; Memory, Attention and Decision Processes	5.1 Information about health consequences; 12.1 Restructuring physical environment; 1.4 Action planning; 4.1 Instruction on how to perform behaviour; 2.2 Feedback on behaviour; 7.1 Prompts/cues	Inform about health consequences (BCIO:007063); Restructure the physical environment (BCIO:050348); Action planning (BCIO:007010); Instruct how to perform a behaviour (BCIO:007058); Provide feedback on behaviour (BCIO:007023) Prompt intended action (BCIO:007080)	Improved diagnostic tools (expanded‐range culture, molecular testing); Clinician education on SUC false‐negative rates and test limitations; Decision aids supporting symptom‐based assessment alongside test results; Clear rUTI care pathways with referral criteria; Longer appointments for complex/recurrent presentations; Shared care records across services; System prompts for proactive follow‐up
*Unheard voices*	*“Because they see UTIs every day, they just don't think it's that important.”* (Bea) *“I've never had a doctor who has even considered the idea of a false negative. They just believe in the test and not me.”* (Sophie) *“You always get told that it's not there and that maybe you're just stressed. It's that connection to women being slightly hysterical.”* (Zara) *“Definitely kind of dumbing down of my pain to an extent. ‘You can't be in that much pain, just go home and take paracetamol.’”* (Dalia) *“You don't even get a phone call, it [the online patient portal] just goes ‘negative result,’ and that shuts the conversation down.”* (Anna)	Social Influences; Emotion; Beliefs about Consequences; Social/Professional Role and Identity	3.3 Social support (emotional); 5.1 Information about health consequences; 6.2 Social comparison; 8.1 Behavioural practice/rehearsal; 11.2 Reduce negative emotions; 9.1 Credible source; 4.3 Re‐attribution	Deliver emotional support (BCIO:007041); Arrange emotional support (BCIO:007036); Awareness of other people's thoughts, feelings and actions (BCIO:007072) Inform about health consequences (BCIO:007063); Practise behaviour (BCIO:007094); Present information from credible influence (BCIO:007075); Re‐attribute cause (BCIO:007053)	Clinician education on diagnostic test limitations in rUTI; Training on symptom‐led assessment; Integration of symptom severity and quality of life assessment into consultations; Implicit bias awareness training; Communication skills for diagnostic uncertainty; Education on rUTI quality of life impact using patient‐reported outcomes data; Communication skills training on validation and active listening
*Shouldering blame*	*“The thing that frustrates me the most is always, always being told by doctors, by pharmacists, by friends and family, the first thing is always, ‘oh, well, you're not weeing after sex.”* (Ellie) *“It's almost like they're trying to blame you, you know, ‘do you wipe from front to back? Do you do this? Do you do that?’ And, you know, it's like teaching your mum to suck eggs.”* (Julie) *“I don't want to be blamed for drinking coffee, because it's got absolutely nothing to do with coffee.”* (Nicole) *“Every time I've gone in, it's always with the same vein of, ‘well, if you decrease sex,’ or ‘are you in a honeymoon period or anything else?’ It's the immediate bias that is towards sex or something I've done in regards to sex. You're instantly shamed.”* (Dalia) *“You can feel a little bit judged, you know, they kind of say, ‘oh, it's poor hygiene.”* (Rachel)	Social Influences; Beliefs about Consequences; Emotion; Social/Professional Role and Identity	5.1 Information about health consequences; 13.2 Framing/reframing; 3.3 Social support (emotional); 11.2 Reduce negative emotions; 6.2 Social comparison; 15.3 Focus on past success	Inform about health consequences (BCIO:007063); Reframe past behaviour (BCIO:007056); Suggest different perspective on behaviour (BCIO:007302); Deliver emotional support (BCIO:007041); Arrange emotional support (BCIO:007036); Prompt focus on past success (BCIO:007139)	Reframe rUTI aetiology (away from hygiene/behaviour); Reduce blame‐oriented questioning; Case studies demonstrating harm of psychological dismissal; Mental health screening and support pathways; Non‐judgemental communication training; Reframe from inevitability to manageable condition; Peer support signposting; Emotional wellbeing assessment in consultations; Safety‐netting protocols; System prompts for review appointments; Expand NHS access to specialist investigations, treatments
*Forced to become an expert*	*“I'd say probably in the last 10/15 years, the doctors have let me treat myself. I just get repeat prescriptions. I check my UTIs myself. For example, I buy my own urinalysis dipsticks.”* (Tracy) *“Nobody's trying to help you link up the pieces, so unless you are pushing to link up the pieces yourself, nobody's going to do it for you.”* (Anna) *“No one will ever express an opinion now in the health service, which I understand, but it also makes it really hard, because you ask which is better, and they say, ‘well, it's really up to you.’ Well, gee, thanks.”* (Karen) *“It's a job in itself to manage your condition. It actually possibly only improved because I've taken those steps myself, it's not advice I've been given.”* (Nicole) *“I really fought to get that ultrasound. I wouldn't leave the practice.”* (Charlotte)	Environmental Context and Resources; Skills; Behavioural Regulation; Beliefs about Capabilities; Memory, Attention and Decision Processes	12.1 Restructuring physical environment; 3.2 Social support (practical); 1.4 Action planning; 1.2 Problem solving; 4.1 Instruction on how to perform behaviour; 15.1 Verbal persuasion about capability; 11.1 Pharmacological support	Restructure the physical environment (BCIO:050348); Deliver instrumental support (BCIO:007040); Action planning (BCIO:007010); Instruct how to perform a behaviour (BCIO:007058); Arrange emotional support (BCIO:007036); Persuade about personal capability (BCIO:007137); Provide pharmacological support (BCIO:007145)	Reduce system burden requiring patient coordination; Validated self‐management resources; Care coordinator support for complex cases; Shared care records reducing repetition; Patient advocacy support/services; Clinician training on responding to informed patients
FACILITATORS					
*Feeling validated*	*“When they do listen, I just really feel like you're a bit more respected as a human.”* (Sian) *“He said, ‘what can we do for you?’ I said, ‘I just want you to listen.’“* (Julie) *“She's the first person I've actually felt believed by and heard. She was thinking about me as a person, that this isn't just my bladder, it's lifestyle, it's social things, it's my stress. She supported my whole body.”* (Alexandra) *“I was just a number in it all, like, there was no real understanding of, like, the pain and discomfort that I was going through.”* (Sian) *“Actually listening to me without being on a time schedule or trying to tick the boxes. Just talking to someone is just incredible.”* (Jean)	Social Influences; Emotion; Social/Professional Role and Identity; Beliefs about Consequences	3.3 Social support (emotional); 6.1 Demonstration of behaviour; 8.1 Behavioural practice/rehearsal; 11.2 Reduce negative emotions; 6.2 Social comparison; 13.5 Identity associated with changed behaviour	Deliver emotional support (BCIO:007041); Arrange emotional support (BCIO:007036); Demonstrate the behaviour (BCIO:007055); Practise behaviour (BCIO:007094); Adopt changed self‐identity (BCIO:007160)	Active listening and communication skills training; Protected consultation time; Validate patient knowledge as useful diagnostic information; Patient experience feedback; Empathy and compassion training; Holistic assessment incorporating wellbeing and quality of life; Professional identity as patient‐centred practitioner
*Partners in the puzzle*	*“I think it's important for patients to feel like they're having some control in an area where sometimes that control is taken away.”* (Rachel) *“I know my body, I know what I'm going to react to.”* (Anna) *“He said, that's our gold star treatment and he drew me the little diagram and explained. He was trying to show us the evidence.”* (Karen) *“[Having a question list] almost feels like someone else has got your back.”* (Sian) *“I think like a tracking app would be fantastic for UTIs. It could give a whole record of your behaviour and your treatment that would enable you to put together a picture of what might particularly be a trigger for you. You'd be able to present it to the doctor more readily.”* (Zara)	Social Influences; Goals; Intentions; Knowledge; Beliefs about Capabilities	1.4 Action planning; 1.1 Goal setting (behaviour); 5.1 Information about health consequences; 9.2 Pros and cons; 1.9 Commitment; 1.2 Problem solving; 3.2 Social support (practical)	Action planning (BCIO:007010); Set behaviour goal (BCIO:007003); Inform about health consequences (BCIO:007063); Consider pros and cons (BCIO:007069); Change behaviour goal (BCIO:050445); Goal strategising (BCIO:007008); Awareness of other people's thoughts, feelings and actions (BCIO:007072) Deliver practical social support (BCIO:007040)	rUTI‐specific shared decision‐making training; Patient decision aids for treatment options; Written care plans with contingencies (Plan A, B, C); Explanation of treatment rationale; Scheduled follow‐up; Clinician commitment to shared decision‐making
*Continuity and connection*	*“I think it's the continuity, otherwise, it feels like every time you're going in, you're explaining the situation again.”* (Sian) *“I guess now, because those new guidelines have said that a pharmacist can do it. It's good because at least that's less of a treachery than trying to get a GP appointment.”* (Anna) *“If you're face‐to‐face with someone, like, there's an extra level of investment from both sides. We get to read the body language and things like that, it's an extra layer of communication.”* (Charlotte) *“I always take someone with me [to appointments]. Someone with me would never have let someone speak to me like that.”* (Naomi) *“What's really useful is case studies and video case studies. It's peer to peer stuff.”* (Paul)	Environmental Context and Resources; Social Influences; Memory, Attention and Decision Processes; Reinforcement	12.1 Restructuring physical environment; 7.1 Prompts/cues; 3.2 Social support (practical); 2.7 Feedback on outcome of behaviour; 6.2 Social comparison; 10.8 Incentive (outcome)	Restructure the physical environment (BCIO:050348); Deliver instrumental support (BCIO:007040); Prompt intended action (BCIO:007080); Provide feedback on outcome of behaviour (BCIO:007027); Awareness of other people's thoughts, feelings and actions (BCIO:007072); Arrange emotional support (BCIO:007036);	Named clinician continuity; Longer appointments for complex presentations; Shared care records to maintain history; Pre‐appointment information gathering to maximise time; Multiple access routes (pharmacy, OOH, virtual); Clear guidance on when to access which service; Peer support network signposting; Bringing support person to appointments encouraged
*Expanding the toolkit*	*“If I think about the d‐mannose and Hiprex, that was me finding out about it. Why? Why is that? Why doesn't a GP say, ‘right, okay, so you're getting these recurrent infections.’“* (Anna) *“I'd really like to know what the alternatives are and have conversations around preventative approaches. Knowing what the options are depending on their particular scenario.”* (Maya) *“An understanding that it can look very different for different patients, and that not everybody fits in a neat and tidy box.”* (Rachel) *“Nobody's ever told me the downside of taking one antibiotic a day.”* (Karen) *“He said, ‘oh, don't worry, you can take [antibiotics] for the rest of your life.’ And then I spoke to the other woman, and she said, ‘well, of course, there's always a danger with antibiotics.’“* (Julie)	Knowledge; Beliefs about Consequences; Optimism; Skills; Environmental Context and Resources	11.1 Pharmacological support; 5.1 Information about health consequences; 9.2 Pros and cons; 4.1 Instruction on how to perform behaviour; 12.5 Adding objects to environment; 2.4 Self‐monitoring of outcomes	Provide pharmacological support (BCIO:007145); Inform about health consequences (BCIO:007063); Consider pros and cons (BCIO:007069); Instruct how to perform a behaviour (BCIO:007058); Add objects to the environment (BCIO:007156); Self‐monitor outcome of behaviour (BCIO:007025)	Education on full rUTI toolkit (methenamine hippurate, vaccines, d‐mannose); Decision aids presenting multiple options; Shared learning on treatment success stories; Balanced AMR discussions (not fear‐based); Individual resistance profile reviews; Self‐monitoring tools for treatment outcomes; Preventive approach discussions

*Note:* A thematic map of barriers and facilitators is available in Figure 1, and a theoretical framework illustrated in Figure 2. Recommendations represent practical translations of mapped BCTs and BCIO classes for clinical implementation. For example, BCT 5.1 (information about health consequences) translates to recommendations for clinician education on diagnostic test limitations; BCT 12.1 (restructuring physical environment) informs recommendations for improved diagnostic tools and clearer care pathways. Recommendations were grounded not only in the BCW mapping process but also in participants’ expressed priorities and concerns, as identified through the qualitative analysis, and are consistent with existing qualitative literature on patient experiences of healthcare for chronic conditions including rUTI and related urological conditions (e.g., Bey et al. [17]; Brown et al. [58]; Scott et al. [33], van Horrik et al. [54]).

Abbreviations: BCIO, behaviour change intervention ontology [43, 44]; BCT, behaviour change technique [40, 41, 42]; TDF, theoretical domains framework [39].

### 3.1 Barrier Themes

#### 3.1.1 Struggling With the System

Participants experienced the healthcare system as poorly configured for recurrent UTI, describing unreliable diagnostic tests, rigid clinical guidelines, fragmented services, prolonged waiting times, and marked geographical inequities. Care pathways were experienced as unclear and inconsistent, meaning access to quality rUTI care seemed to depend on luck rather than coordinated, evidence‐based pathways.

Standard diagnostic tools were widely criticised as inaccurate and inconsistent with lived experience, with incongruent results generating confusion about the perceived unreliability of the system:

They did a dip test which showed white blood cells, and then the culture came back ‘nothing.’ You start thinking ‘maybe I'm making it up.’ I just don't understand.

(Sian)

Clinical guidelines were perceived as rigid and narrow, applied to justify treatment refusal without further discussion: *“They just said ‘NICE guidelines’ like that was the end of the conversation. There was no room to discuss my symptoms or history”* (Anna). The absence of clear rUTI care pathways left participants navigating an unpredictable system:

The NHS is all about pathways, and I feel that with UTIs, you're so not on a pathway. It's like a ghastly assortment of rocks and clumps. You stumble your way forward, and you fall over, and then you're back at the beginning.

(Karen)

rUTI healthcare was experienced as reactive rather than longitudinal, with consultations focused on resolving individual UTI episodes rather than managing recurrence. Several participants desired a more long‐term, strategic approach to rUTI management:

He honestly didn't do anything. He just said, ‘carry on with [the current treatment].’ That was it. No plan. No follow‐up. It was so rushed. It just felt like [he believed] ‘it's just a UTI.’

(Tracy)

Fragmentation across siloed services had emotional and practical consequences. Several participants reported being discharged or lost to follow‐up despite ongoing symptoms, with Paul describing feeling “forgotten about” by the healthcare system. One participant described how conflicting clinician approaches left her without treatment and severe, escalating symptoms:

There was a crossover between my GP and urology, and they were arguing back and forth, so then I didn't get any antibiotics for a month. I've ended up in A&E twice because of this, hospitalised with IV antibiotics because the flare was so bad and I couldn't access medication.

(Scarlet)

However, participants also commonly perceived there to be marked inconsistency in clinician knowledge, particularly regarding rUTI‐specific management, emerging treatments, and approaches to antibiotic prescribing. While some clinicians were perceived as overly cautious due to antimicrobial stewardship concerns, others prescribed more readily. Geographical variation further compounded these issues, with rUTI care widely described as a “postcode lottery.” The cumulative effect was a sense that quality care depended on luck:

It's like Russian roulette. No two people have told me the same thing, and it's the inconsistency that breaks your trust the most.

(Lina)

#### 3.1.2 Unheard Voices

Participants described feeling dismissed, doubted, or silenced in clinical encounters. Credibility was shaped by power, identity, and communication style, with gender, ethnicity, and familiarity with medical language influencing whether symptoms were taken seriously.

Reliance on diagnostic tests over patient‐reported symptoms was prominent, with negative results overriding embodied knowledge and visible symptoms:

They were taking my urine samples and then calling me and saying, ‘You haven't got a UTI, it's coming back clear.’ I was weeing blood, and they were like, ‘no, no, no.’

(Scarlet)

Symptoms were frequently reframed as psychological, with several participants describing clinicians who had attributed them to stress or even suggested they were false or exaggerated:

I was 100% certain that I had a UTI. I'm very in tune with my body, but when I went to see him, he just said to me, ‘I just think it's psychosomatic. It's all in your head. Just go home and relax.’

(Jean)

The severity and impact of rUTI were frequently trivialised, with clinicians framing infections as minor inconveniences rather than conditions warranting attention. For some, repeated dismissal eroded self‐trust:

It got to the point that I then started thinking, maybe they're right [about UTIs not being important]. When the people you trust are telling you these things, you think, ‘is it in my head?’

(Karen)

Credibility also appeared to vary by gender. Several female participants perceived that males with rUTI were treated differently: *“If men got UTIs like women did, it'd be totally different. If a male gets it, it's a different ball game”* (Tracy). Indeed, Paul reported a contrasting experience: *“I don't feel disbelieved, no. They [the doctors] were willing to have a go.”*

In addition, those with professional healthcare backgrounds or familiarity with medical language reported being taken more seriously:

When you use the proper names, the medical terminology, they take you more seriously.

(Anna)

Racial bias was also explored, with Dalia describing how her ethnicity shaped clinical assumptions:

Immediately they went, ‘you're diabetic, you're Afro‐Caribbean.’ I do have to advocate for myself more, but if I advocate too much, I have the opposite effect and I'm seen as difficult, so I'm kind of stuck in the middle.

(Dalia)

Across these accounts, participants felt their experiences were systematically discounted within a hierarchy that prioritised diagnostic results over embodied knowledge and patient‐reported symptoms.

#### 3.1.3 Shouldering Blame

rUTI was frequently individualised and moralised, with people living with rUTI shouldering blame for their recurrent symptoms through narratives emphasising hygiene practices, sexual behaviour, and gendered normalisation of symptoms. This framing fostered stigma while minimising the perceived need for clinical investigation or long‐term management.

Many participants reported feeling blamed for their infections, describing the cumulative effect of repeated behavioural questioning:

It'll always be a case of like, ‘well, you could be doing these things,’ like, ‘are you wiping back to front? Have you peed after sex?’ I've done all of that. At this point, I'd have to be extremely negligent to be getting them at the rate I'm getting them. I feel like I'm blamed quite a lot.

(Dalia)

This sense of blame became internalised for many: *“It feels like there is almost a bit of blame in the condition, that I'm doing something wrong*” (Ellie).

Participants reflected on feeling patronised and disrespected, with advice overly focused on sexual behaviour experienced as particularly distressing:

The first thing that I was told was, ‘well, you just weren't cleaning up after,’ and you know, ‘you should just stop having sex.’ I was shouted at during that time, and it was really unpleasant, and I cried.

(Alexandra)

rUTI was also framed as an inevitable, unremarkable part of life, particularly for women – a form of implicit blame that positioned recurrence as something people living with rUTI should simply accept. This normalisation minimised suffering and reduced clinical urgency:

I think they just think it's an inevitable part of being a woman, and it doesn't have to be. It's just seen as something that will happen to you, rather than something that can actually be treated and prevented.

(Bea)

Such framing narrowed clinical attention to treating individual episodes of UTI symptoms, overlooking psychological and emotional consequences. Yet, the psychological consequences of sustained blame and dismissal were severe. Tracy described the impact at its most extreme: *“At 21, I was seriously thinking of suicide. It totally rips your life apart.”* Such accounts underscored how feeling blamed and responsible compounded the already significant psychological burden of living with rUTI.

The cumulative effect of perceived blame – whether explicit questioning of hygiene practices or implicit normalisation – was a sense that rUTI was participants' own fault to bear, rather than a legitimate medical condition warranting clinical attention and investigation.

#### 3.1.4 Forced to Become an Expert

In the absence of reliable guidance and continuity, participants were compelled to research, self‐test, self‐treat, and manage their rUTI care independently. This work extended beyond medical tasks into emotional and administrative effort, with people living with rUTI co‐ordinating care pathways that participants felt should have been system‐supported.

Critically, this transition into rUTI self‐management was not described as empowerment, but as a necessary survival response to gaps in system coordination and clinical knowledge:

I've had to learn myself, and yeah, I've sort of been my own doctor. For example, when I went to my private urologist, he said something. I said, ‘oh, are you going to put me on methenamine hippurate?’ He went, ‘yeah, how do you know about that?’ Well, I've done a lot of research.

(Naomi)

Several participants described the burden of self‐management and trial‐and‐error treatment:

It does feel a bit like, ‘oh well, oh dear. We'll throw the kitchen sink at you in the hope that something will work’, right? And in that kitchen sink, I've had to kind of pull out the bits that I'm okay with and adjust it to fit me. It's just not okay, you can't live with it.

(Karen)

Accessing quality rUTI care required persistent self‐advocacy, with participants frequently using language of fighting and battling: Anna observed: *“You really have to fight your corner. It's only because I stand up for myself and I shove the NICE guidelines down their face that they vaguely listen to me. If you were not au fait with the NICE guidelines, I imagine your experience would be even worse than mine.”* This highlighted how effective navigation of rUTI healthcare systems depended on resources such as knowledge, confidence, and health literacy that not all people living with rUTI possessed equally. Yet this enforced expertise came at a significant personal cost:

Sometimes you just want to be told what it is you should do, because you've got decision fatigue. My head's in an absolute swell of information, I would just really like someone to tell me what to do.

(Rachel)

For some, the burden of navigating challenging rUTI care within the NHS system extended to financing their own treatment, exacerbating any pre‐existing financial challenges related to reduced work ability while living with chronic illness:

I'm living off a pension, so I could sustain private [healthcare] for a short while, but definitely not long term. I'm having to give up work because of this condition.

(Jean)

While participants demonstrated remarkable resourcefulness in developing personalised management strategies, this expertise emerged from necessity rather than choice, reflecting the burden of self‐directed care due to perceived gaps in system support.

### 3.2 Facilitator Themes

#### 3.2.1 Feeling Validated

Feeling listened to and believed was described as profoundly meaningful, restoring trust, dignity, and emotional safety. Validation of lived experience signalled legitimacy and demonstrated a shift from distress to therapeutic engagement.

Being given attention and space to share created emotional safety and helped participants articulate what living with rUTI really entails. When clinicians asked questions that considered the breadth of rUTI impact, participants felt understood and that their condition was taken seriously. Clinicians acknowledging symptoms as real, rather than contingent on test results, validated participants and made them feel credible. The emotional weight of finally feeling believed was evident:

Just feeling like someone believes what I've got, you know, it's quite powerful. [participant pauses] Sorry, I don't know why I'm crying.

(Sophie)

Compassionate communication, empathy, and respect helped repair confidence that had been broken down during previous rUTI healthcare experiences. Participants recalled encounters where clinicians *“actually seemed interested”* (Nicole) and treated them *“like a human being and not just a number”* (Michael), contrasting clearly with experiences of dismissal:

When a clinician is empathetic, for me, it validates that I'm not, like, doing something wrong in being there. Them kind of showing some empathy or validating you just makes you feel like you're not wasting anyone's time.

(Sophie)

Validation was particularly powerful when clinicians took on a more holistic view, recognising the broader impact of rUTI on quality of life, relationships, work and daily activities, and emotional wellbeing rather than focusing narrowly on test results. Feeling validated enabled participants to re‐engage with rUTI care from a place of safety rather than defensiveness.

#### 3.2.2 Partners in the Puzzle

Collaborative care enabled people living with rUTI to participate meaningfully in decisions, with both lived experience and prepared knowledge valued within consultations. When clinicians were receptive to participants' embodied expertise, tracked history, and independent research, participants described feeling more confident and less uncertain. Being involved in decisions fostered a much‐wanted sense of control and agency:

You're in charge of your destiny, I know it sounds cheesy, but the person that knows my body best is me. Being an engaged member of your own decision making, it's your own body, and you don't get a decision in it. It's crap. Even in, you know, just the choice of antibiotic, at least it gives you some control.

(Maya)

Clear explanations and reasoning for treatment decisions increased trust in clinicians and their expertise, reducing uncertainty with rUTI care and treatment. Sian described what effective SDM looked like: “*It was a shared decision‐making process. He kind of laid out the options and explained the success rates. He recommended trying this route first because of the lack of side effects.”*

Care plans and longer‐term strategising reassured participants that clinicians were considering their futures. When clinicians worked with participants to map out options, it fostered a sense of shared ownership over rUTI management. This partnership approach, characterised by presenting options, explaining rationales, and planning contingencies, enabled participants to feel like active collaborators rather than passive recipients of care:

I think if clinicians can lay out a scenario of, ‘we could try this for 3 months, but then if that didn't work, we'd have Plan B, which could be this or this.’ It gives you a little bit of insight.

(Rachel)

Participants valued opportunities to bring knowledge into consultations, both the embodied expertise of knowing their own bodies and symptoms, and prepared information such as questions, notes, tracked history, and independent research. When clinicians were receptive, this expertise and preparation supported communication, confidence, and better rUTI care: *“I came with my research and she actually listened and said, ‘that's really helpful’”* (Elizabeth). Partnership was experienced as active collaboration, where both clinical and experiential knowledge of rUTI were acknowledged as legitimate and valuable.

#### 3.2.3 Continuity and Connection

Connected care was created through sustained clinician relationships and access to support beyond the clinic. People living with rUTI described continuity, social support, and flexible routes into rUTI care as protective, contextualised against feelings of being abandoned or alone.

Having continuity in working with the same clinicians enabled symptom patterns to emerge, thorough histories to be collated, and participants to feel greater trust in their judgements:

Seeing the same person helps. They know your history, they can see the pattern. I didn't have to go back to the beginning.

(Christine)

Longer appointment durations created space for deeper engagement and stronger relationship building. Adequate time enabled the bigger picture‐building that participants valued:

The best appointment I've had was with [private doctor], like, realising that I wasn't the only one, and just him kind of talking about what options I could have, in depth as well, not just kind of a quick, rushed appointment.

(Bea)

Support beyond clinical environments also played a critical role, with participants finding strength in not facing rUTI alone. Peer communities offered recognition, validation, and shared knowledge:

I was talking to a friend at the weekend who has [a UTI] at the moment, and I mean, I loved the fact I could sit there and talk to her for ages about this issue. I was sharing my learnings with her, and was like, ‘oh, have you tried this? Have you tried that?'

(Ellie)

Bringing family or friends to appointments also provided practical support, emotional back‐up, and someone to advocate alongside them: *“Often I will bring my partner with me, because I feel like they can be less condescending when someone else is there”* (Lina). Continuity, connection, and social support operated together as protective factors against feeling abandoned and uncertain within the rUTI healthcare system.

#### 3.2.4 Expanding the Toolkit

Access to non‐antibiotic treatments, preventive approaches, and nuanced discussions about AMR gave participants a sense of agency and possibility. Personalised management replaced one‐size‐fits‐all prescribing and enabled rUTI care beyond crisis response.

Options beyond antibiotics, including methenamine hippurate, vaccines, and d‐mannose, gave hope to people living with rUTI. When options were presented and tailored to individual circumstances and histories, they felt their specific needs were being considered:

She talked me through the different options. It wasn't just ‘oh, here's another antibiotic.’

(Sian)

Several participants reflected on the frustration that these options had existed but were not offered earlier: *“I think Hiprex [methenamine hippurate] has massively helped, but in hindsight, it's frustrating that I've gone so many years without any of these options being offered to me”* (Bea). This underscored both the value of expanded options and the need for clinicians to be aware of the full therapeutic toolkit.

Participants expressed desire for conversations about alternatives to antibiotic treatment, given anxiety about resistance. Meaningful discussions about AMR balanced risk with the reality that people living with rUTI often require antibiotics:

He explained about resistance but also said ‘we need to treat you properly.’ It wasn't just about saying no.

(Nicole)

A broader therapeutic toolkit transformed rUTI care from reactive to proactive, and from uncertain to actionable.

### 3.3 Theoretical Mapping

Systematic mapping revealed all 14 TDF domains were implicated across themes, with ‘social influences,’ ‘beliefs about consequences,’ ‘environmental context and resources’ and ‘knowledge’ most frequently represented. Table 3 presents the complete mappings from themes through TDF domains, BCTs, and BCIO classes to recommendations.

The most frequently identified BCTs were: 5.1 information about health consequences, 3.3 social support – emotional, 12.1 restructuring physical environment, and 4.1 instruction on how to perform behaviour. Each BCT links to specific BCIO classes specifying the intervention mechanism. For instance, BCT 3.3 maps to BCIO:007041 (deliver emotional support) and BCIO:007036 (arrange emotional support). Recommendations represent accessible clinical translations of these BCT‐BCIO pairings, elaborated on within the four priority intervention areas in the Discussion.

## 4 Discussion

This study provides the first application of the Theoretical Domains Framework (TDF) and behaviour change theory to patient experiences of rUTI healthcare, enabling systematic identification of behavioural determinants and theory‐informed intervention targets [39, 40, 41, 42, 43, 44]. Four barrier and four facilitator themes revealed challenges experienced as systematic at contextual and relational levels, while demonstrating that replicable positive healthcare experiences occur when specific conditions exist.

### 4.1 Summary of Findings

Barrier themes revealed how people living with rUTI experience significant challenges when navigating healthcare for their condition. Participants described a clinical environment characterised by diagnostic uncertainty, rigid guideline application, and fragmented service coordination. Such experiences were compounded by perceived clinical dismissal, particularly when test results contradicted their lived experience of symptoms. People living with rUTI felt they shouldered blame for their illness through narratives of hygiene, behaviour, and gendered normalisation. The final barrier theme captured how people living with rUTI were compelled to research, coordinate, and advocate for their own care, representing necessary adaptation to perceived gaps in coordinated care rather than empowerment. Critically, facilitator themes demonstrated that quality rUTI care is achievable within existing structures. When clinicians listened and explicitly believed people living with rUTI, valued their input in collaborative decision‐making, maintained continuity, and demonstrated knowledge with access to expanded treatment options, experiences clearly shifted. Facilitators were not simply the absence of barriers, but distinct positive conditions that transformed rUTI care. The theoretical framework (Figure 2) illustrates how contextual and relational factors shape the responses of people living with rUTI into either exhausting self‐advocacy or supported self‐management and partnership.

### 4.2 Theoretical Contributions

This study makes several key contributions to knowledge. It provides the first theoretically grounded analysis of patient experiences of rUTI healthcare, demonstrating the utility of the TDF for identifying behavioural determinants that underpin both negative and positive experiences. The analysis also revealed that facilitators are not simply the absence of barriers but represent distinct enabling conditions. This suggests intervention development should actively create facilitating conditions, not solely remove barriers. Finally, the findings offer a replicable methodology for translating qualitative patient experience data into actionable, theory‐informed intervention targets. Such methodology is applicable to other chronic conditions within urology [58, 59], and others characterised by diagnostic uncertainty and credibility challenges such as fibromyalgia and chronic fatigue syndrome [60, 61, 62, 63].

All 14 TDF domains were implicated across the barrier and facilitator themes, indicating that improving rUTI healthcare requires both system‐level restructuring (e.g., pathways, continuity, resources) and interpersonal skill enhancement (e.g., validation, communication, SDM). The mapping of themes to TDF domains enabled systematic identification of BCTs, BCIO classes, and theory‐informed recommendations (Table 3). This approach provides a robust foundation for developing and evaluating complex interventions targeting rUTI healthcare quality.

The combination of RTA [45] with the Behaviour Change Wheel (BCW) [40] framework represents a methodological strength. RTA's commitment to researcher reflexivity and iterative approach to creating meaning ensured themes remained grounded in participant experiences [45, 46, 47], while the BCW's systematic approach to identifying behavioural determinants and intervention techniques enabled translation of experiential insights into actionable targets for service improvement [40, 41, 42, 43]. This combined approach demonstrates how patient experience research can valuably inform intervention development.

### 4.3 Clinical Implications and Priority Areas for Intervention

Synthesis of themes and mappings to the TDF domains, BCTs, and BCIO identified four priority areas with direct implications for clinical practice and service development.

Firstly, addressing diagnostic understanding and clinical flexibility is essential given the centrality of test‐symptom mismatch to experiences of dismissal. Clinician education on the documented limitations of dipstick and SUC testing [18, 19, 20, 21, 22, 23], combined with decision aids supporting symptom‐based assessment, could help align clinical practice with patient experiences [11]. Development of improved diagnostic tools may further address this disconnect [21, 64, 65].

Secondly, strengthening validation and patient‐centred communication could address the finding that test results were consistently prioritised over lived experience. Integration of validated patient‐reported outcome measures (PROMs), such as the Recurrent UTI Symptom Scale (RUTISS) and the Recurrent UTI Impact Questionnaire (RUTIIQ) [13, 66, 67], offers a practical mechanism for capturing symptom severity and quality of life within consultations, providing structured data to inform decision‐making and intervention evaluation. Communication and implicit bias training could help replicate empathetic clinical encounters that participants found transformative.

Thirdly, embedding collaborative care and SDM into routine practice and rUTI management could systematically reproduce positive experiences. Training supported by decision aids presenting the full range of therapeutic options, alongside patient‐facing tools such as question prompt lists and symptom trackers, would enable the partnership approach that increased confidence and trust and reduced uncertainty. Structured care plans with contingencies would address the desire for longer‐term strategising rather than reactive management of UTI episodes.

Finally, improving system coordination and continuity would reduce the burden currently falling on people living with rUTI. Named clinician continuity, shared detailed patient histories, and clearer rUTI care pathways could enable enhanced relationship building and pattern recognition. Expanding clinician knowledge of and patient access to non‐antibiotic options, including methenamine hippurate, UTI vaccines, and intravesical instillations, offers a patient‐centred approach to antimicrobial stewardship while providing evidence‐supported alternatives [22, 68, 69, 70, 71, 72, 73].

### 4.4 Strengths and Limitations

This study provides the first in‐depth qualitative exploration of rUTI healthcare experiences within the UK NHS context, including GP systems, NICE guidelines, and pharmacy‐based UTI pathways. While this focus provided depth, the core findings regarding diagnostic uncertainty, perceived dismissal, and the importance of patient validation likely have broader relevance, with international research documenting similar challenges [5, 9, 17]. The theoretical framework and recommendations may therefore have wider applicability with adaptation beyond local healthcare contexts. Strengths of this study include application of established theoretical frameworks enabling actionable recommendations and partnership with key rUTI stakeholders ensuring experiential validity.

Limitations include the predominantly female, White, and degree‐educated sample, limiting transferability to underrepresented populations. Although findings suggested potential gender differences in credibility and treatment, conclusions are limited given fewer male participants. Participants were predominantly recruited online through key UTI stakeholder groups, rather than clinical services. However, given established limitations of standard diagnostic testing and recommendations to prioritise patient‐report [18, 19, 20, 21, 22, 23], this approach appropriately centred patient‐identified experiences and facilitated access to broader representation from across the UK. Further, while the UK focus enables contextual depth, it is acknowledged that this may limit generalisability. While qualitative sample sizes are typically small, lies in elucidating contextual mechanisms and lived experiences that shape care delivery. These findings therefore offer essential insight into the processes underpinning population‐level patterns in rUTI healthcare [45, 46, 47, 51].

### 4.5 Future Research

Several research directions follow from these findings. Firstly, studies with more diverse samples are essential, including greater representation of males living with rUTI, those identifying as non‐binary, minority ethnic groups, and those from more varied socioeconomic backgrounds. Although this study does not assess the prevalence of identified experiences, it identifies mechanisms and service‐level barriers relevant to population‐level improvement. Evaluation of theory‐informed interventions across diverse settings will be essential to determine how effectively these mechanisms can be modified in practice and to ensure that service redesign reduces, rather than reinforces, inequities. Secondly, intervention studies are a key next step to develop and evaluate the identified theory‐informed recommendations, particularly PROM integration, SDM training, and non‐antibiotic pathway education and access. The APEASE criteria should be applied to ascertain implementation feasibility within resource‐constrained primary care services [40]. The Template for Intervention Description and Replication (TIDieR) checklist should be employed during reporting to ensure completeness and replicability [74]. Health economic evaluation would support implementation by demonstrating value alongside effectiveness. Thirdly, the recommendations presented in this paper should be viewed as theory‐informed targets for future intervention development; further work is required to co‐design, pilot, and evaluate specific interventions in practice. Finally, qualitative research with rUTI healthcare professionals is necessary to understand clinician‐perceived barriers and facilitators, enabling interventions that address challenges on both sides of the consultation.

## 5 Conclusion

This study identified barriers to quality rUTI healthcare perceived as systematic at both contextual and relational levels, with people living with rUTI forced to become experts when these barriers dominate. Facilitator themes demonstrate that validation, collaboration, continuity, and expanded treatment knowledge and options transform experiences when present. The Theoretical Domains Framework identifies four priority intervention areas: diagnostic understanding and clinical flexibility, validation and patient‐centred communication including PROM integration, collaborative care and SDM, and system coordination and continuity. When people living with rUTI feel believed, involved, and supported, healthcare experiences are fundamentally shifted. These findings demonstrate that quality rUTI care is not aspirational but achievable; implementing the conditions that facilitate it must now become a priority.

## Author Contributions

All authors contributed to study conceptualisation and methodology, including design of the interview schedule. Abigail F. Newlands and Melissa L. Kramer contributed to resources and recruitment of participants. Abigail F. Newlands managed the study administration, conducted data collection and investigation, and completed data curation and initial formal data analysis. All authors contributed to formal analysis and review of qualitative themes. Abigail F. Newlands prepared the original draft of the article, and all authors reviewed and approved the final article. Sarah Snuggs and Katherine A. Finlay supervised the project.

## Funding

The authors received no specific funding for this work.

## Ethics Statement

This study received ethical approval from the University of Reading Research Ethics Committee (reference: UREC 24/17). All participants provided informed e‐consent before interviews, were advised that their participation was confidential, optional, and could be withdrawn at any time without giving a reason. Pseudonyms have been employed throughout the manuscript and personal identifying information removed to maintain participant confidentiality.

## Patient or Public Contribution

This study was conducted in collaboration with Live UTI Free (https://liveutifree.com/), a leading UTI patient research and advocacy organisation. Melissa L. Kramer (MLK), CEO of Live UTI Free, is a researcher and patient expert with personal lived experience of rUTI. Abigail F. Newlands (AFN) is a researcher with personal lived experience of UTI and related urogynaecological health conditions. Katherine A. Finlay (KAF) is a health psychology researcher and chartered health psychologist, also with personal lived experience of recurrent UTI. AFN, MLK, KAF, and other patient experts at Live UTI Free co‐developed the interview schedule with the research team, ensuring questions captured the breadth of concerns and experiences of people living with recurrent UTI. AFN, MLK, and KAF also contributed to study design, facilitated participant recruitment through Live UTI Free networks, participated in analytical discussions throughout theme development, and contributed to article preparation. This partnership ensured the research was grounded in authentic patient priorities.

## Conflicts of Interest

Melissa L. Kramer is the CEO of Live UTI Free Ltd.; however, no financial incentives have been received.

## Supporting information

Supporting Information S1: Qualitative Reporting Checklists (RTARG & BQQRG).

Supporting Information S2: Full interview schedule & Theoretical Domains Framework.

## Data Availability

Due to the sensitive nature of the qualitative research and ethical agreements, full transcripts are not shared openly. Anonymised data summaries and quotations are available within the article. Further information and access to de‐identified datasets can be requested from the corresponding author.
