# Supplementary material for: From Dismissal to Partnership: Patient Experiences of Recurrent Urinary Tract Infection Healthcare Informed by the Theoretical Domains Framework and Behaviour Change Theory
Source: Health Expect. 2026 Mar 1;29(2):e70629. doi: 10.1111/hex.70629 (PMC12949963; doi:10.1111/hex.70629)
Supplement: Supplementary file 2 — Supporting Information S2: Full interview schedule & Theoretical Domains Framework. [file HEX-29-e70629-s002.docx]

**Full interview schedule (pp. 1-5) and the Theoretical Domains Framework (pp. 6-8)**

**Interview schedule:**

| **Questions and probes** | **TDF domain(s)** |
| --- | --- |
| ***Introduction*** |  |
| 1. To start off, please can you describe your history with recurrent urinary tract infections and the healthcare you have experienced for this condition. |  |
| - 1. Please describe a recent medical appointment you attended for your recurrent UTI.      1. NHS/private, general/specialist      2. Ease of discussion      3. Rapport, familiarity (regular doctor?)      4. Willingness to listen      5. Ability to ask questions      6. Feeling heard and listened to      7. Ease of access |  |

| ***We’re now going to talk about the communication between people living with recurrent UTI and medical professionals.*** |  |
| --- | --- |
| 1. Can you tell me what you feel makes good communication possible when you are attending medical appointments for recurrent UTI? | - Skills - Social/professional role and identity - Beliefs about capabilities - Beliefs about consequences - Optimism - Environmental context and resources - Social influences - Intentions - Goals - Emotion - Behavioural regulation |
| - 1. To what extent do you feel good communication is important for you when living with recurrent UTI? |  |
| - 1. What does open communication mean to you within your medical appointments? |  |
| 1. During a medical appointment for recurrent UTI, what do you feel are the most important topics to discuss? | - Memory, attention and decision processes - Skills - Goals |
| - 1. Can you tell me about a time when these topics have been covered *well* in your own appointments? |  |
| - 1. Can you tell me about a time when these topics have been covered *badly* in your own appointments? |  |
| 1. Thinking of clinicians and the ways they interact with you in medical appointments for recurrent UTI, what do you find most helpful? Why? | - Skills - Social/professional role and identity - Optimism - Environmental context and resources - Social influences |
| - 1. What do you find the least helpful? Why? |  |
| - 1. What skills do you think clinicians should hold or develop to communicate better with people living with recurrent UTI? |  |

| 1. As the person receiving medical care, can you tell me about how the ways you may communicate may affect the appointment in a positive way? | - Knowledge - Social/professional role and identity - Skills - Beliefs about consequences - Reinforcement |
| --- | --- |
| - 1. And negatively? |  |
| - 1. Please could you describe what a helpful appointment with good communication might be like for you?      1. How might things have to change for that vision to become possible?      2. What things might have to change? | - Environmental context and resources - Social influences - Knowledge - Intentions - Goals - Reinforcement |
| 1. Thinking about the environment within which you attend medical appointments for your recurrent UTI, what do you feel are the factors that encourage helpful communication? | - Environmental context and resources - Reinforcement |

| ***We’re now going to move on to talk about getting involved in the medical decisions made about your recurrent UTI.*** |  |
| --- | --- |
| 1. Thinking back to a recent medical appointment you attended for your recurrent UTI, to what extent do you feel you shared in the decision-making process with your doctor or other healthcare professional? | - Memory, attention and decision processes - Beliefs about capabilities - Social/professional role and identity |
| 1. What does it mean to you when you are able to play an active role within your UTI medical care?    - 1. How does this impact you and/or your perception of the medical appointment / your relationship with your doctor / emotions?      2. Could you describe an example of playing an active role within your UTI medical care? | - Beliefs about capabilities - Beliefs about consequences - Emotion - Behavioural regulation - Social/professional role and identity |
| 1. What, if anything, do you do to prepare for your UTI medical appointments?    - 1. Plans      2. Goals      3. Confidence in attaining goals? | - Intentions - Goals - Behavioural regulation - Optimism - Knowledge |
| 1. Can you reflect on how your preparation affects your experience during the medical appointment, if at all?    - 1. Emotions      2. Treatment decisions      3. Patient satisfaction      4. Doctor-patient relationship      5. Feeling listened to, etc. | - Memory, attention and decision processes - Knowledge - Goals - Emotion - Beliefs about consequences - Behavioural regulation |

| 1. Can you tell me about any factors that you feel may hinder or act as barriers to you getting involved in the medical decisions made about your recurrent UTI? | - Knowledge - Skills - Social/professional role and identity - Beliefs about capabilities - Beliefs about consequences - Optimism - Environmental context and resources - Social influences - Intentions - Goals - Emotion - Behavioural regulation |
| --- | --- |
| - 1. Clinician factors |  |
| - 1. Personal/individual factors |  |
| - 1. Social factors |  |
| - 1. Other, e.g., environmental factors |  |
| ***We’re going to move on to the final topic now. We’re thinking about how challenges in UTI care could be improved.*** |  |
| 1. If we were to develop a resource or tool to improve patient-doctor communication in recurrent UTI, what do you think it should include? Why? | - Memory, attention and decision processes - Skills - Social/professional role and identity - Knowledge - Reinforcement |
| - 1. When do you think the optimum time would be to use such a resource or tool? |  |
| - 1. How would you use it? |  |
| - 1. For patients and/or clinicians? |  |
| - 1. How could positive habits be encouraged through a resource or tool like this? |  |
| 1. Is there anything else you would like to add? |  |

**Key references**

Cane, J., O’Connor, D., & Michie, S. (2012). Validation of the theoretical domains framework for use in behaviour change and implementation research. *Implementation Science*, *7*(1), 37. <https://doi.org/10.1186/1748-5908-7-37>

Huijg, J. M., Gebhardt, W. A., Crone, M. R., Dusseldorp, E., & Presseau, J. (2014). Discriminant content validity of a theoretical domains framework questionnaire for use in implementation research. *Implementation Science*, *9*(1), 11. <https://doi.org/10.1186/1748-5908-9-11>

**Theoretical Domains Framework (from Cane et al., 2012):**

| **Domain (definition)** | **Constructs** |
| --- | --- |
| 1. **Knowledge** (An awareness of the existence of something) | - Knowledge (including knowledge of condition/scientific rationale) - Procedural knowledge - Knowledge of task environment |
| 1. **Skills** (An ability or proficiency acquired through practice) | - Skills - Skills development - Competence - Ability - Interpersonal skills - Practice - Skills assessment |
| 1. **Social/Professional Role and Identity**   (A coherent set of behaviours and displayed personal qualities of an individual in a social or work setting) | - Professional identity - Professional role - Social identity - Identity - Professional boundaries - Professional confidence - Group identity - Leadership - Organisational commitment |
| 1. **Beliefs about Capabilities** (Acceptance of the truth, reality, or validity about an ability, talent, or facility that a person can put to constructive use) | - Self-confidence - Perceived competence - Self-efficacy - Perceived behavioural control - Beliefs - Self-esteem - Empowerment - Professional confidence |

| 1. **Optimism** (The confidence that things will happen for the best or that desired goals will be attained) | - Optimism - Pessimism - Unrealistic optimism - Identity |
| --- | --- |
| 1. **Beliefs about Consequences** (Acceptance of the truth, reality, or validity about outcomes of a behaviour in a given situation) | - Beliefs - Outcome expectancies - Characteristics of outcome expectancies - Anticipated regret - Consequents |
| 1. **Reinforcement** (Increasing the probability of a response by arranging a dependent relationship, or contingency, between the response and a given stimulus) | - Rewards (proximal/distal, valued/not valued, probable/improbable) - Incentives - Punishment - Consequents - Reinforcement - Contingencies - Sanctions |
| 1. **Intentions** (A conscious decision to perform a behaviour or resolve to act in a certain way) | - Stability of intentions - Stages of change model - Transtheoretical model and stages of change |
| 1. **Goals** (Mental representations of outcomes or end states that an individual wants to achieve) | - Goals (distal/proximal) - Goal priority - Goal/target setting - Goals (autonomous/controlled) - Action planning - Implementation intention |
| 1. **Memory, Attention and Decision Processes** (The ability to retain information, focus selectively on aspects of the environment and choose between two or more alternatives) | - Memory - Attention - Attention control - Decision making - Cognitive overload/tiredness |
| 1. **Environmental Context and Resources** (Any circumstance of a person’s situation or environment that discourages or encourages the development of skills and abilities, independence, social competence, and adaptive behaviour) | - Environmental stressors - Resources/material resources - Organisational culture/climate - Salient events/critical incidents - Person x environment interaction - Barriers and facilitators |
| 1. **Social influences** (Those interpersonal processes that can cause individuals to change their thoughts, feelings, or behaviours) | - Social pressure - Social norms - Group conformity - Social comparisons - Group norms - Social support - Power - Intergroup conflict - Alienation - Group identity - Modelling |
| 1. **Emotion** (A complex reaction pattern, involving experiential, behavioural, and physiological elements, by which the individual attempts to deal with a personally significant matter or event) | - Fear - Anxiety - Affect - Stress - Depression - Positive/negative affect - Burnout |
| 1. **Behavioural Regulation** (Anything aimed at managing or changing objectively observed or measured actions) | - Self-monitoring - Breaking habit - Action planning |
